# Analysis of *BRCA1*and *BRCA2* large genomic rearrangements in Sri Lankan familial breast cancer patients and at risk individuals

**DOI:** 10.1186/1756-0500-7-344

**Published:** 2014-06-06

**Authors:** Sumadee De Silva, Kamani Hemamala Tennekoon, Eric Hamilton Karunanayake, Indrani Amarasinghe, Preethika Angunawela

**Affiliations:** 1 Institute of Biochemistry, Molecular Biology and Biotechnology, University of Colombo, Colombo, Sri Lanka; 2 National Cancer Institute, Colombo, Sri Lanka; 3 Department of Pathology, Faculty of Medicine, University of Colombo, Colombo, Sri Lanka

**Keywords:** *BRCA1*, *BRCA2*, Large genomic rearrangements, Breast cancer

## Abstract

**Background:**

Majority of mutations found to date in the *BRCA1*/*BRCA2* genes in breast and/or ovarian cancer families are point mutations or small insertions and deletions scattered over the coding sequence and splice junctions. Such mutations and sequence variants of *BRCA1* and *BRCA2* genes were previously identified in a group of Sri Lankan breast cancer patients. Large genomic rearrangements have been characterized in *BRCA1* and *BRCA2* genes in several populations but these have not been characterized in Sri Lankan breast cancer patients.

**Findings:**

A cohort of familial breast cancer patients (N = 57), at risk individuals (N = 25) and healthy controls (N = 23) were analyzed using multiplex ligation-dependent probe amplification method to detect *BRCA1* and *BRCA2* large genomic rearrangements. One familial breast cancer patient showed an ambiguous deletion in exon 6 of *BRCA1* gene. Full sequencing of the ambiguous region was used to confirm MLPA results. Ambiguous deletion detected by MLPA was found to be a false positive result confirming that *BRCA1* large genomic rearrangements were absent in the subjects studied. No *BRCA2* rearrangement was also identified in the cohort.

**Conclusion:**

Thus this study demonstrates that *BRCA1* and *BRCA2* large genomic rearrangements are unlikely to make a significant contribution to aetiology of breast cancer in Sri Lanka.

## Findings

### Background

Germ-line mutations in *BRCA1* and *BRCA2* tumor suppressor genes cause a hereditary predisposition to breast and ovarian cancer [1]. Such mutations account for 15-20% of familial breast cancer [2]. Although familial breast cancer contributes to 5-10% of all breast cancers, individuals carrying mutations in one of these genes have a 40-80% chance of developing breast cancer [3]. At present, in Sri Lanka women are diagnosed with breast cancer at a median age of 50 years contributing to approximately 25% of all cancers [4]. Majority of mutations found to date in the *BRCA1*and *BRCA2* genes in breast and/or ovarian cancer families are point mutations or small insertions and deletions scattered over the whole coding sequence and the splice junctions. Point mutations and sequence variants in *BRCA1*[5] and *BRCA2*[6] genes were previously identified in this cohort of Sri Lankan breast cancer patients. More recently, large genomic alterations have been described in *BRCA1* and *BRCA2* genes. Such large alterations lead to change in genomic copy number and cannot be detected by conventional methods [7]. Rearrangements have occasionally been reported in patients who are negative for *BRCA1/BRCA2* mutations [8].

There is a difference in the degree of *BRCA1/BRCA2* rearrangements found in different ethnic groups and populations. The prevalence of *BRCA1/BRCA2* genomic rearrangements in Asians is thought to be low. However studies done in these populations are limited. Several deletions and duplications have been reported from Singapore [9], Korea [8], Malaysia [10] and China [11]. There are no published reports on the analysis of *BRCA1* and *BRCA2* large genomic rearrangements in Sri Lankans and this study examined the possibility of such genomic rearrangements in a cohort in which point mutations and sequence variants in *BRCA1* and *BRCA2* were previously described [5,6].

### Results and discussion

**Figure 1:**
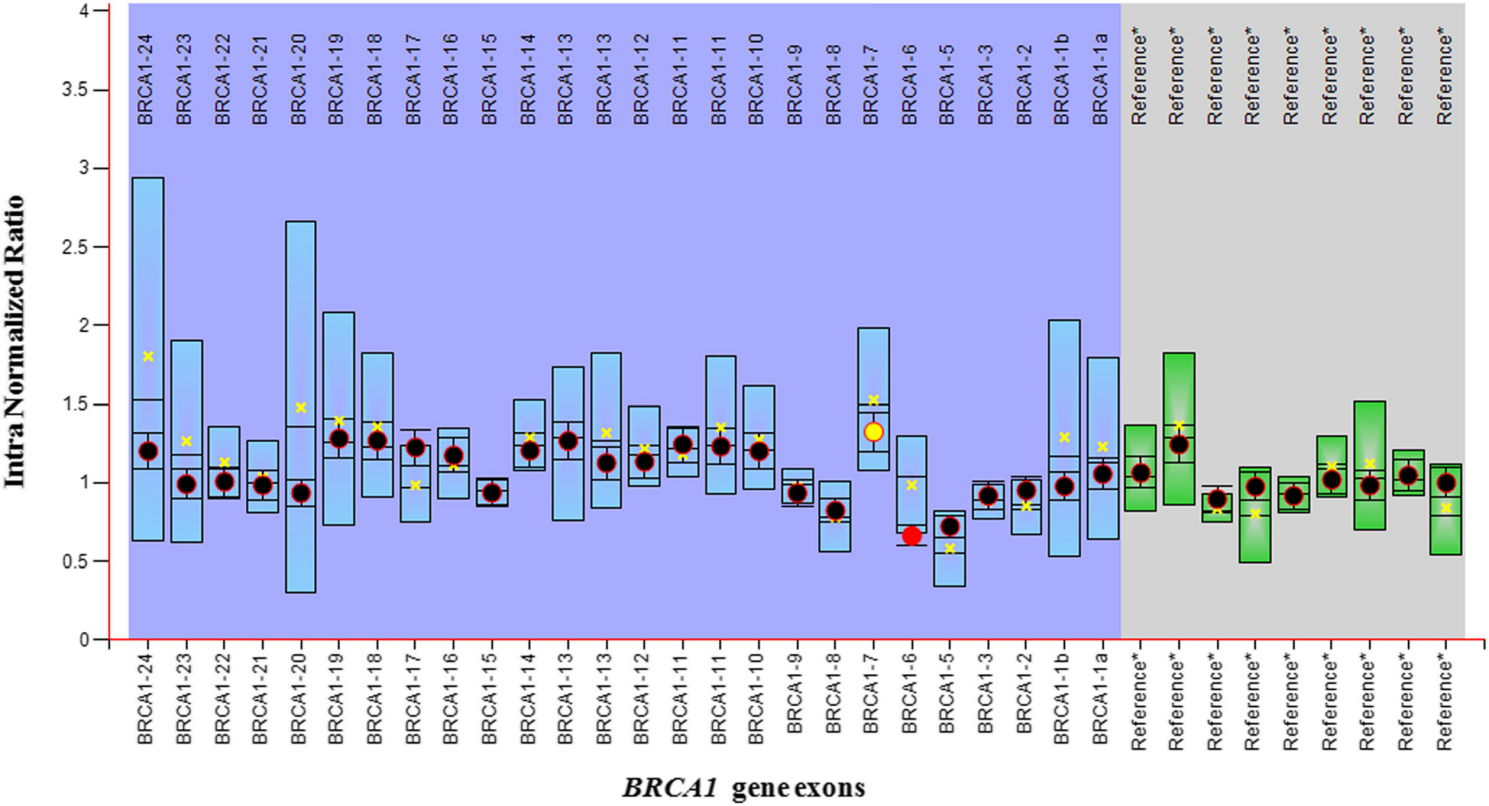
**MLPA results for *BRCA1 *gene.** MLPA results for *BRCA1* gene of a patient with familial breast cancer in bar chart format generated by Coffalyser*.*Net 01 software. *BRCA1* exons and intra normalized ratio are given on the X axis and Y axis respectively (intra normalized ratio: division of probe signal of each amplification product by all reference probe signals within the run). Probe ratios are indicated by the dots. Black: within the 95% confidence interval (CI) of the reference sample population, Red: out of the 95% CI and over the arbitrary borders (0.7 to 1.3 by default), Yellow: within the 95% CI, but over the arbitrary borders. Whiskers: 95% CI for sample value (test or reference). Boxes: 95% CI in reference sample population (by default). Blue: compared to test probes, Green: compared to reference probes.

**Figure 2:**
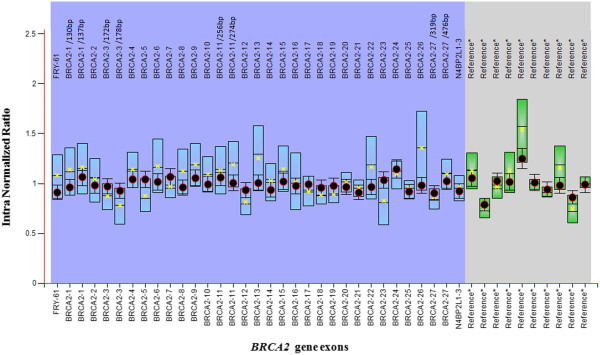
**MLPA results for *BRCA2 *gene.** MLPA results for *BRCA2* gene of a patient with familial breast cancer in bar chart format generated by Coffalyser*.*Net 01 software. *BRCA2* exons and intra normalized ratio are given on the X axis and Y axis respectively (intra normalized ratio: division of probe signal of each amplification product by all reference probe signals within the run). Probe ratios are indicated by the dots. FRY- 61 probe and N4BP2L1-3 probe detect sequences just before and after the *BRCA2* gene. Black: within the 95% confidence interval (CI) of the reference sample population, Red: out of the 95% CI and over the arbitrary borders (0.7 to 1.3 by default), Yellow: within the 95% CI, but over the arbitrary borders. Whiskers: 95% CI for sample value (test or reference). Boxes: 95% CI in reference sample population (by default). Blue: compared to test probes, Green: compared to reference probes.

MLPA analysis did not reveal any large genomic rearrangements in *BRCA2* gene in any of the subjects studied. However, according to MLPA analysis, one breast cancer patient was detected with an average intra normalized ratio (probe signal of each amplification product/ all reference probe signals within each sample run) of 0.64 in exon 6 of *BRCA1* gene which was predicted as an ambiguous deletion. An average ratio of 0.64 indicates a reduction in relative peak area of the amplification product by 36%. Figure 1 shows the MLPA results of the patient found to have an ambiguous exon 6 deletion for *BRCA1*. Figure 2 shows the MLPA results for the *BRCA2* gene of one of the familial breast cancer patients. The average ratios were within the 0.7-1.3 range indicating absence of exon deletions or duplications of the *BRCA2* gene in all samples analysed.

In the patient who showed an ambiguous deletion for *BRCA1* exon 6, the average ratio for exon 6 was outside the 95% confidence limits and outside the arbitrary range of 0.7 to 1.3. Although for exon 7, the average ratio exceeded the arbitrary range this was still within the 95% confidence limits. Thus we attempted to confirm the *BRCA1*/ exon 6 deletion by direct sequencing. The sample was sequenced (both forward and reverse strands) along with a healthy control sample for comparison. Sequence data were analysed using Mutation Surveyor DNA Variant Analysis Software – Softgenetics against reference sequence of *BRCA1* in the basic local alignment search tool (BLAST) published by National Centre for Biotechnology Information (NCBI), USA (accession no. L78833). Sequence data showed no change in the DNA sequence of exon 5, 6 and 7of the patient compared to reference sequence. Data also indicated that the MLPA probe hybridization site was intact.

Familial breast cancer patients and at risk individuals were previously investigated for *BRCA1* and *BRCA2* mutations and some were positive for *BRCA1/2* mutations. From this cohort, three at risk individuals and two familial breast cancer patients were positive for clearly pathogenic *BRCA1* mutations and only six familial breast cancer patients were positive for clearly pathogenic *BRCA2* mutations. The same cohort was used for the detection of *BRCA1* and *BRCA2* genomic rearrangements in the present study.

Detection of *BRCA* rearrangements is very important in a population since in some populations the occurrence of large deletions and duplications in either *BRCA1* or *BRCA2* is substantial. A prevalence of 2.1% for *BRCA1* large genomic rearrangements has been detected in Spanish hereditary breast/ovarian cancer families testing negative for point variations and small insertions/deletions in *BRCA1* and *BRCA2*[12]*. BRCA1/2* large genomic rearrangements have shown noticeable founder effect in certain European and American populations. One large genomic rearrangement, *BRCA1* exon 9–12 deletion, is considered as a mutation in Mexican population [13]. In Northern Finland, a large deletion of exons 1A-13 in *BRCA1* gene is currently identified to represent 14.3% (1/7) of the Finnish population [14].

*Arthrobacter luteus* (*Alu)* short stretches of repetitive DNA appear to be the main source of large genomic rearrangements by providing hotspots for unequal homologous recombination [15,16]. Several large genomic rearrangements reported in *BRCA1* have been frequently recognized within intragenic *Alu* repeats [16] and *BRCA1* pseudogene (Ψ*BRCA1)* 30 kb upstream [17,18]. To date, at least 81 different large genomic rearrangements have been found in *BRCA1* gene and account for 8%–27% of all *BRCA1* mutations. Alternatively *Alu* sequences are less common in *BRCA2* gene, where only few large genomic rearrangements are reported, accounting for 0%–11% of all *BRCA2* mutations [19-21].

The aim of this study was to assess the contribution of *BRCA1*and *BRCA2* rearrangements for predisposing to breast cancer in familial breast cancer patients and at risk individuals in Sri Lanka. We did not observe any conclusive large genomic rearrangements of *BRCA1* and *BRCA2* among the subjects studied. However in other Asian countries like Singapore three novel *BRCA* rearrangements have been found [9]. These were exon 13 duplication and exon 13–15 deletion of *BRCA1,* and exon 4–11 duplication in *BRCA2. BRCA1* genomic rearrangement found in Korean population involved exons 13–15. This exon 13–15 deletion has also been identified in three families with America/French-German, Danish, and Singaporean/Indian ethnicities [12,22,23]. In a Malaysian population, two genomic rearrangements in *BRCA1* (exon 13–15 deletion and exon 1–14 deletion) and one in *BRCA2* (exon 14–16 deletion) were detected [10].

According to the findings of the present study *BRCA1* and *BRCA2* large genomic rearrangements are unlikely to significantly contribute to breast cancer in Sri Lanka. This is the first report on the analysis of *BRCA1* large genomic rearrangements in Sri Lanka.

The importance of recognizing the large rearrangements with respect to *BRCA1/2* is explained by *BRCA* rearrangements showing apparent founder effect in some populations which can be used as diagnostic tools. BRAC Analysis Large Rearrangement Test (BART) is already established in the country like US and also has been introduced as new updates to HBOC (Hereditary Breast and Ovarian Cancer) guidelines by National Comprehensive Cancer Network (NCCN). So BART is especially recommended for the individuals with strong personal and family history of breast and ovarian cancer along with routine BRCA analysis. Under such circumstances, it is so important to undergo large genomic rearrangements analysis by the familial breast cancer patients and their at risk individuals in a particular population as a diagnosis tool for breast cancer.

### Conclusion

Although we failed to find any conclusive *BRCA1* large genomic rearrangement and did not find any *BRCA2* large genomic rearrangement in familial breast cancer patients and at risk individuals in the current study, a large study sample especially including Eurasians and other ethnic groups may reveal novel or reported genomic rearrangements among Sri Lankans.

### Methods

#### Study participants

A total of 105 participants (N = 57 with a family history of breast cancer, N = 25 at risk individuals and N = 23 healthy controls without a personal or family history of any cancer) were studied. Mean age at diagnosis was 47.76 ± 9.55 years for familial breast cancer patients.

Fourteen familial patients were diagnosed below 40 years of age. Mean age at the MLPA analysis was 36.88 ± 14.95 for at risk individuals. Among the familial cases 34, 17 and 4 patients had one, two and three affected family members respectively. Two patients had 4 affected family members. According to histopathlogical data of familial breast cancer patients, 48 had infiltrating (invasive) ductal carcinoma and data were not recorded for remaining 9 patients. None of the patients had metastasis.

The majority of the patients and controls and all at-risk individuals were ethnically Sinhalese. There were no descendents of Europeans. Ethical approval from the Research, Ethics and Higher Degree Committee, Institute of Biochemistry, Molecular Biology and Biotechnology, University of Colombo and written informed consent from the study participants were obtained prior to the study.

Genomic DNA was extracted using the protocol described by Miller et al. [24] from aliquots of peripheral blood samples that had been stored at -20˚C. MLPA was performed using the SALSA MLPA KIT P002-C1 BRCA1 probemix and SALSA MLPA KIT P090 BRCA2 probemix (MRC-Holland, Amsterdam, Netherlands) for *BRCA1*and *BRCA2* genes according to manufacturer’s protocol. The processed data obtained via MegaBACE Genetic Profiler software suite*®* v2.2 as well as via ABI GeneMapper*®* v4.1 were analyzed by using Coffalyser*.*Net 01 software.

Exon 5, 6 and 7 specific primers were designed in order to confirm the predicted ambiguous deletion detected from MLPA data. These primers were able to amplify whole regions of exon 5, 6 and 7 and MLPA probe hybridization site as well as several intronic regions. Resultant PCR products were subjected to direct sequencing using Applied Biosystems™ 3500 DX Genetic Analyzer in order to locate the deletion site of exon 6 as well as to confirm the data obtained from MLPA analysis.

## Competing interest

Authors declare that they have no competing interests.

## Authors’ contribution

SDS carried out molecular genetic studies, sequence alignment and drafted the manuscript. EHK and KHT conceived and designed the study, helped molecular genetic studies, data analysis and revision of the manuscript. IA and PA provided clinical expertise, recruitment of study participants and supervised clinical data and sample collection. All authors read and approved the final manuscript.

## References

[B1] Martin AM Blackwood MA Antin-Ozerkis D Shih HA Calzone K Colligon TA Seal S Collins N Stratton MR Weber BL Nathanson KL Germline mutations in BRCA1 and BRCA2 in breast-ovarian families from a breast cancer risk evaluation clinic J Clin Oncol 2001 19 2247 2253 11304778 10.1200/JCO.2001.19.8.2247

[B2] Peto J Cancer epidemiology in the last century and the next decade Nature 2001 411 390 395 10.1038/35077256 11357148

[B3] Fackenthal JD Olopade OI Breast cancer risk associated with BRCA1 and BRCA2 in diverse populations Nat Rev Cancer 2007 7 937 948 10.1038/nrc2054 18034184

[B4] Cancer Incidence Data Sri Lanka Year 2001–2005 Cancer Registry National Cancer Control Programme 2009 Colombo 5

[B5] De Silva W Karunanayake EH Tennekoon KH Allen M Amarasinghe I Angunawala P Ziard MH Novel sequence variants and a high frequency of recurrent polymorphisms in *BRCA1* gene in Sri Lankan breast cancer patients and at risk individuals BMC Cancer 2008 8 214 10.1186/1471-2407-8-214 18662409 PMC2519088

[B6] De Silva S Tennekoon KH Karunanayake EH De Silva W Amarasinghe I Angunawela P Novel sequence variants and common recurrent polymorphisms of *BRCA2* in Sri Lankan breast cancer patients and a family with *BRCA1* mutations Exp Ther Med 2011 2 1163 1170 22977638 10.3892/etm.2011.337 PMC3440786

[B7] Mazoyer S Genomic rearrangements in the BRCA1 and BRCA2 genes Hum Mutat 2005 25 415 422 10.1002/humu.20169 15832305

[B8] Seong MW Cho SI Noh DY Han W Kim SW Park CM Park HW Kim SY Kim JY Park SS Low contribution of BRCA1/2 genomic rearrangement to high-risk breast cancer in the Korean population Fam Cancer 2009 8 505 508 10.1007/s10689-009-9279-z 19669600

[B9] Lim YK Lau PT Ali AB Lee SC Wong JE Putti TC Sng JH Identification of novel BRCA large genomic rearrangements in Singapore Asian breast and ovarian patients with cancer Clin Genet 2007 71 331 342 10.1111/j.1399-0004.2007.00773.x 17470134

[B10] Kang P Mariapun S Phuah SY Lim LS Liu J Yoon SY Thong MK Mohd Taib NA Yip CH Teo SH Large BRCA1 and BRCA2 genomic rearrangements in Malaysian high risk breast-ovarian cancer families Breast Cancer Res Treat 2010 124 579 584 10.1007/s10549-010-1018-5 20617377

[B11] Kwong A Ng EK Law FB Wong HN Wa A Wong CL Kurian AW West DW Ford JM Ma ES Novel BRCA1 and BRCA2 genomic rearrangements in Southern Chinese breast/ovarian cancer patients Breast Cancer Res Treat 2012 136 931 933 10.1007/s10549-012-2292-1 23099436 PMC3511694

[B12] de la Hoya M Gutiérrez-Enríquez S Velasco E Osorio A Sanchez de Abajo A Vega A Salazar R Esteban E Llort G Gonzalez-Sarmiento R Carracedo A Benítez J Miner C Díez O Díaz-Rubio E Caldes T Genomic rearrangements at the BRCA1 locus in Spanish families with breast/ovarian cancer Clin Chem 2006 52 1480 1485 10.1373/clinchem.2006.070110 16793929

[B13] Torres D Rashid MU Seidel-Renkert A Weitzel JN Briceno I Hamann U Colombian Breast Cancer Study Group (COLBCS) Absence of the BRCA1 del (exons 9–12) mutation in breast/ovarian cancer families outside of Mexican Hispanics Breast Cancer Res Treat 2009 117 679 681 10.1007/s10549-009-0383-4 19333752

[B14] Pylkas K Erkko H Nikkila J Solyom S Winqvist R Analysis of large deletions in *BRCA1*, *BRCA2* and *PALB2* genes in Finnish breast and ovarian cancer families BMC Cancer 2008 8 146 10.1186/1471-2407-8-146 18501021 PMC2413256

[B15] Deininger PL Batzer MA Alu repeats and human disease Mol Genet Metab 1999 67 183 193 10.1006/mgme.1999.2864 10381326

[B16] Smith TM Lee MK Szabo CI Jerome N McEuen M Taylor M Hood L King MC Complete genomic sequence and analysis of 117 kb of human DNA containing the gene *BRCAI* Genome Res 1996 6 1029 1049 10.1101/gr.6.11.1029 8938427

[B17] Puget N Gad S Perrin-Vidoz L Sinilnikova OM Stoppa-Lyonnet D Lenoir GM Mazoyer S Distinct *BRCA1* rearrangements involving the *BRCA1* pseudogene suggest the existence of a recombination hot spot Am J Hum Genet 2002 70 858 865 10.1086/339434 11880951 PMC379114

[B18] Montagna M Santacatterina M Torri A Menin C Zullato D Chieco-Bianchi L D'Andrea E Identification of a 3 kb Alu-mediated BRCA1 gene rearrangement in two breast/ovarian cancer families Oncogene 1999 18 4160 4165 10.1038/sj.onc.1202754 10435598

[B19] Stadler ZK Saloustros E Hansen NA Schluger AE Kauff ND Offit K Robson ME Absence of genomic BRCA1 and BRCA2 rearrangements in Ashkenazi breast and ovarian cancer families Breast Cancer Res Treat 2010 123 581 585 10.1007/s10549-010-0818-y 20221693

[B20] Sluiter MD van Rensburg EJ Large genomic rearrangements of the *BRCA1* and *BRCA2* genes: review of the literature and report of a novel *BRCA1* mutation Breast Cancer Res Treat 2011 125 325 349 10.1007/s10549-010-0817-z 20232141

[B21] Mahon SM Large genomic rearrangements in *BRCA1* and *BRCA2*. Implications for patient care Oncol Nurs Forum 2013 40 220 222 10.1188/13.ONF.220-222 23615136

[B22] Thomassen M Gerdes AM Cruger D Jensen PK Kruse TA Low frequency of large genomic rearrangements of BRCA1 and BRCA2 in Western Denmark Cancer Genet Cytogenet 2006 168 168 171 10.1016/j.cancergencyto.2005.12.016 16843109

[B23] Gad S Caux-Moncoutier V Pages-Berhouet S Gauthier-Villars M Coupier I Pujol P Frénay M Gilbert B Maugard C Bignon YJ Chevrier A Rossi A Fricker JP Nguyen TD Demange L Aurias A Bensimon A Stoppa-Lyonnet D Significant contribution of large BRCA1 gene rearrangements in 120 French breast and ovarian cancer families Oncogene 2002 21 6841 6847 10.1038/sj.onc.1205685 12360411

[B24] Miller SA Dykes DD Poleskey HF A simple salting out procedure for extracting DNA from human nucleated cells Nucleic Acids Res 1988 16 12 15 10.1093/nar/16.3.1215 PMC334765 3344216

